# Mechanism of Magnolia Volatile Oil in the Treatment of Acute Pancreatitis Based on GC-MS, Network Pharmacology, and Molecular Docking

**DOI:** 10.1155/2023/3503888

**Published:** 2023-02-07

**Authors:** Shengmao Li, Yu Huang, Lin Liu, Fan Zhang, Hui Ao, Yingping Luo

**Affiliations:** ^1^School of Pharmacy, North Sichuan Medical College, Nanchong 637007, China; ^2^Department of Clinical Medicine, North Sichuan Medical College, Nanchong 637007, China; ^3^College of Pharmacy, Chengdu University of Traditional Chinese Medicine, Chengdu 611137, China

## Abstract

**Objective:**

*Magnoliae officinalis* cortex (MOC) is one of the most frequently used traditional Chinese medicine (TCM) for the treatment of acute pancreatitis (AP). Magnolia volatile oil (MVO) is considered to be one of the main active ingredients in MOC for AP treatment. However, the underlying mechanism of MVO in AP therapy is unknown.

**Methods:**

An integrated strategy of gas chromatography-mass spectrum (GC-MS), network pharmacology, and molecular docking simulation was employed to predict underlying mechanism of MVO in AP treatment. First, the compounds of MVO were identified by GC-MS, and the targets of the identified characteristic compounds were collected from several databases, as well as AP-related targets. Next, Gene Ontology (GO) and Kyoto Encyclopedia of Genes and Genomes (KEGG) analysis were carried out to obtain the mechanism. Moreover, the binding activity between core therapeutic targets and their corresponding compounds was evaluated by molecular docking simulation.

**Results:**

GC-MS results showed a total of 35 compounds that appeared in at least 18 out of 20 chromatograms were considered as characteristic compounds of MVO, and 33 compounds of those were identified. Network analysis demonstrated that 33 compounds regulated 142 AP-related targets. Of those, 8 compounds (*α*-eudesmol, *γ*-eudesmol, (−)-terpinen-4-ol, terpineol, hinesol, linalool, borneol, and *β*-eudesmol) and 8 targets (TNF, IL-1*β*, PPAR*γ*, PPAR*α*, PTGS2, NCOA1, CNR1, and ESR1) have a close relationship with AP treatment and were recognized as the key active compounds and the core therapeutic targets, respectively. The 142 targets were involved in both inflammation and calcium overload-related biological pathways, such as neuroactive ligand-receptor interaction, estrogen, MAPK, and calcium signaling pathway. Moreover, molecular docking simulation indicated that the 8 core therapeutic targets strongly interacted with their corresponding compounds.

**Conclusions:**

In summary, the present study elucidated that the efficacy of MVO in AP treatment might be attributed to anti-inflammation and inhibition of calcium overload through multicomponents and multitargets.

## 1. Introduction

Acute pancreatitis (AP) is the most frequent digestive system illness with both high morbidity and mortality [1, 2]. The overall incidence rate of AP is 4.8–38 per 100,000 annually [3–6] and continually increasing [7, 8]. Moreover, about 20% of AP might progress into severe acute pancreatitis (SAP) [9] due to associated necrosis of the pancreatic tissue and/or multiorgan failure, resulting in as high as 30% of deaths [10]. The pathogenesis of AP is complex and still poorly understood, mainly involving trypsinogen activation, pancreatic microcirculation malfunction, endoplasmic reticulum stress, calcium overload, and inflammatory [8, 11]. The current clinical therapy of AP is mainly symptomatic improvement, such as pain relief and the correction of fluid, electrolyte, or pH-disorders [12]. However, there are no effective drugs available for treating such a complicated disease. Therefore, the development of more effective new drugs is urgently needed for AP patients. As a key part of complementary and alternative medicine, traditional Chinese medicine (TCM), characterized as multicomponents and multitargets, has been confirmed to have a remarkable effect in AP treatment [11, 13, 14].

*Magnoliae officinalis* cortex (MOC), obtained from the dried bark of stem, root, and branches of *Magnolia officinalis* Rehd. et Wils or *Magnolia officinalis* Rehd. et Wils. var. biloba Rehd. et Wils, is one of the most commonly used TCM for treating AP [15–17]. Magnolia volatile oil (MVO), a complex mixture composed of a large number of terpenoids and their oxygenated derivatives, is the main active ingredient of MOC. Plenty of studies indicate that MVO possesses a wide range of bioactivities including anti-inflammatory [18], antibacterial [19, 20], antioxidant, and promoting gastric emptying [21] activities, showing a great potential of clinic application of MVO in AP treatment. Moreover, pieces of evidence suggest that the common compounds in MVO, limonene and borneol, have shown potent anti-AP efficacy in several animal models via antioxidative and anti-inflammatory [22, 23]. However, the key active compounds and underlying mechanism of MVO in AP treatment are not entirely clear, which has driven further investigation.

Network pharmacology is a novel and effective method to systematically and comprehensively describe the law of interactions between components and targets through the integration of chemoinformatics, bioinformatics, network biology, network analysis, and traditional pharmacology [24, 25]. Recently, network pharmacology has been successfully employed to investigate the active compounds and the underlying mechanism of TCM with complex chemical composition [26–30]. Molecular docking simulation is an important way of drug virtual screening, which can provide useful information about drug-receptor interactions by calculating the binding energy of small-molecule ligands (drugs) with proteins (receptors) [31–35]. Recently, molecular docking simulation has been widely used to explore the mechanism of TCM [36–40]. However, the active compounds and the underlying mechanism of MVO in AP treatment were yet to be elucidated based on network pharmacology and molecular docking simulation.

**Figure 1:**
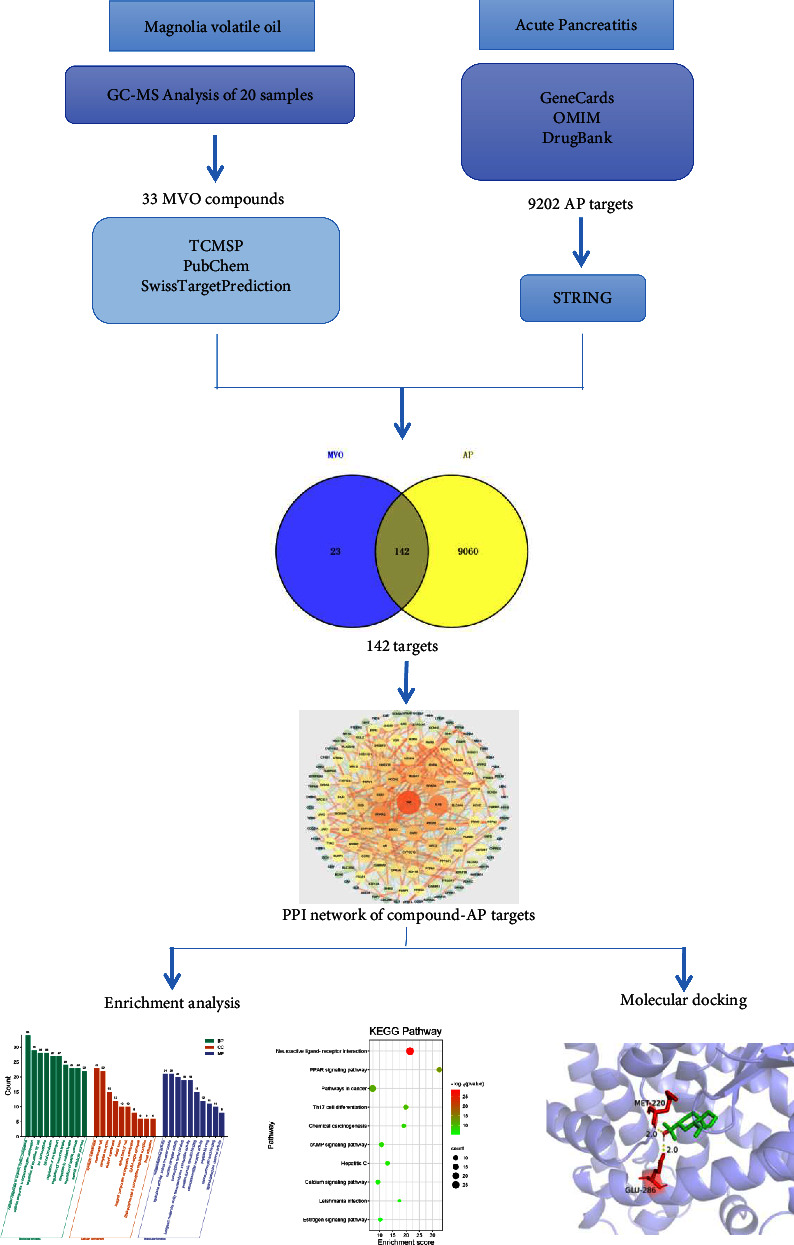
Flowchart of investigating the mechanism of MVO in AP treatment.

As we all know, the chemical composition of TCM is complex and exhibit large differences in the types and quantities as affected by many factors, such as species, geographical regions, harvesting time, and processing conditions [41–45]. This would make it extremely difficult to understand the active compounds and the mechanism of TCM. Though this problem has been alleviated using network pharmacology-based analysis, the data of compounds were only obtained from few known databases, or from the results of chemical composition analyses of a single sample, the difference in chemical composition of TCM caused by differences sources was rarely considered in network pharmacology analysis, which would make the result lack generalizability and representativeness because those compounds cannot effectively reflect the chemical composition features of TCM from various sources. Therefore, in this study, the volatile compounds in MOC from different sources (species and geographical regions) were identified by gas chromatography-mass spectrum (GC-MS), and the mechanisms of MVO in AP treatment were predicted through network pharmacology and molecular docking simulation. A detailed flowchart of the network pharmacology-based study is presented in Figure 1.

## 2. Materials and Methods

### 2.1. Preparation of Sample Solution

A total of 20 batches of MOC were collected from Chengdu Lotus Pond Chinese Herbal Medicine Market (Chengdu, China) and authenticated by associate Professor Lu Chen (Chengdu University of TCM). About 100 g of MOC (dried and powdered) and 1,000 mL of water were put into a volatile oil distillation apparatus (recorded in 2015 Edition Chinese Pharmacopoeia) and extracted by steam distillation for 6 h. The MVO was subsequently collected from the volatile oil extractor and dried by anhydrous sodium sulfate. Twenty milligram of the obtained oil was dissolved in 2 mL of n-hexane and filtered through a 0.22 *µ*m filter, and then the solution was analyzed by GC-MS.

### 2.2. GC-MS Analysis

Analysis of MVO was performed using Agilent 7890A-5975C GC-MS with an HP-INNOWAX capillary column (30 m × 0.25 mm × 0.25 *µ*m). The injection volume was 1 *μ*L with 20 : 1 (v/v) ratio split mode. The carrier gas was helium (99.999% purity, 1 mL/min). The temperature of the injector was set as 280°C. The initial oven temperature was kept at 60°C for 5 min; then, it was gradually raised to 120°C at 10°C/min and to 185°C at 2°C/min and kept for 3 min. Finally, it was raised to 220°C at 8°C/min. The mass spectrometer was operated at 70 eV in full scan mode. The compounds in MVO were identified through both National Institute of Standards and Technology (NIST14) database and literature retrieval. The relative content of each compound in the chromatogram was calculated by an area normalization method.

### 2.3. Prediction of Putative Targets of MVO

According to the abovementioned GC-MS results, the compounds that appeared in at least 18 of 20 chromatograms were selected as characteristic compounds of MVO. The Traditional Chinese Medicine Systems Pharmacology Database (TCMSP, https://tcmsp-e.com/) and PubChem Database (https://pubchem.ncbi.nlm.nih.gov/) were introduced to collect the information of the chemical candidates. Furthermore, the SwissTargetPrediction Database (https://www.swisstargetprediction.ch/) was applied to predict the targets of the characteristic compounds action. Then, all the obtained targets were summarized and reimported into the UniProt Database (https://www.uniprot.org/) to convert them into standard target gene names, and nonhuman and unverified targets were deleted.

### 2.4. Target Prediction of MVO in the Treatment of AP and Construction of the Protein-Protein Interaction (PPI) Network

In order to obtain more comprehensive information of the AP-related targets, a systematic search was conducted in three online databases including the Online Mendelian Inheritance in Man (OMIM, https://www.omim.org/), GeneCards (https://www.genecards.org/), and DrugBank (https://www.drugbank.ca/) using “acute pancreatitis” as a keyword to identify the targets associated with AP. To identify the potential targets responsible for AP treatment, the putative targets of MVO were intersected with the AP-related targets via the Venny 2.1.0 online tool (https://bioinfogp.cnb.csic.es/tools/venny/) [46]. The PPI network of the compound-AP targets was constructed by importing the overlapping targets into STRING database (https://string-db.org/) [47]. The species was set to “*Homo sapiens*,” and the lowest confidence score was set to medium confidence (0.4).

### 2.5. Enrichment Analysis

To better uncover the potential biological processes and pathways of MVO in AP treatment, Gene Ontology (GO) and Kyoto Encyclopedia of Genes and Genomes (KEGG) pathway enrichment analysis were performed by introducing the overlapping targets into Metascape database (https://metascape.org/). In our study, the *p* value was limited to less than 0.05.

### 2.6. Network Construction

To systematically investigate the pharmacological mechanism of the active ingredients in MVO, a sample-compound-target network was constructed by using Cytoscape software (Version 3.7.2). In addition, a compound-target-pathway network was created by linking the pathways (directly relating to AP treatment mechanism), targets, and compounds using the Cytoscape software.

### 2.7. Molecular Docking Simulation

Molecular docking simulation was applied to evaluate the interaction relationships between the core therapeutic targets and their corresponding compounds. The protein crystal structures of each core target and the 3D structures of the compounds were downloaded from the RCSB Protein Data Bank database (PDB, https://www.rcsb.org/) and PubChem database (https://pubchem.ncbi.nlm.nih.gov/), respectively. After pretreatment of ligand (compounds) and receptor (protein crystals) structures, both of them were uploaded to AutoDock 4.0 and AutoDock Tools 1.5.6 for molecular docking. The best docking conformation was picked out following the principles of low energy and reasonable conformation and was visualized using PyMOL.

## 3. Results

### 3.1. Chemical Constituent Analysis of MVO

**Figure 2:**
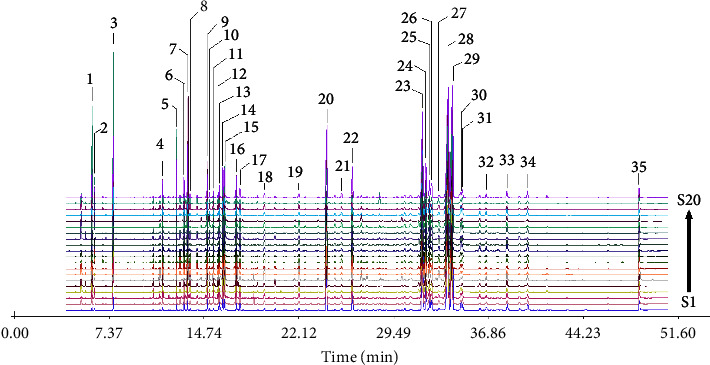
GC-MS chromatogram of 20 batches of *Magnoliae officinalis* cortex (MOC).

The compounds in MVO of 20 samples were identified with GC-MS analysis, and a total of 23 common compounds were detected in the chromatograms of all MVO samples. The 23 common compounds could not perfectly reflect the compounds characteristics of MVO; hence, the sum of their relative content in nearly half of the sample was less than 80%; particularly in S5 and S19, the values were even lower than 65%, was 62.80% and 59.63%, respectively. In an attempt to get more feature information on the compounds in MVO, 35 main compounds appearing in at least 18 of 20 chromatograms were selected as characteristic compounds of MVO, among them 33 compounds were identified. The sum of their relative content was greater than 85% in each sample and consists of 87.51% to 96.41% of the total content. Those 33 compounds could relatively comprehensively reflect the characteristics of the inner main chemical compounds of MVO. Thus, the 33 characteristics compounds were regarded as potential active compounds of MVO in AP treatment and were further used for network pharmacology analysis (Table 1 and Figure 2).

### 3.2. Targets Prediction of MVO in AP Treatment

**Figure 3:**
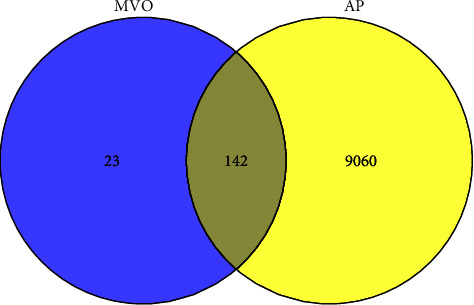
Overlapping targets of MVO and AP.

In our study, a total of 165 targets of 33 potential active compounds abovementioned were obtained through TCSMP, PubChem, as well as SwissTargetPrediction database, and 9202 AP-related targets were collected from several databases including OMIM, GeneCards, and DrugBank database. Furthermore, Venny 2.1.0 was used for Venn analysis, and 142 overlapping targets of MVO in AP treatment were obtained (Figure 3).

### 3.3. Sample-Compound-Target Network

**Figure 4:**
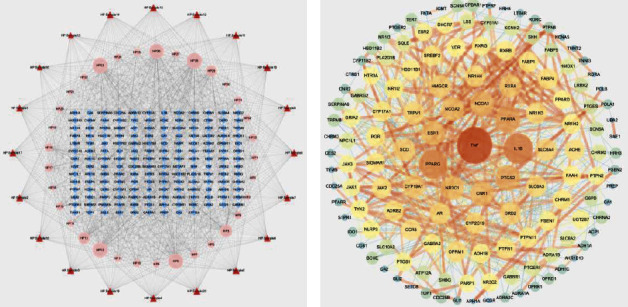
(a) Sample-compound-target network. (b) PPI network of compound-AP targets. The sizes of the nodes are proportional to degree values.

As shown in Figure 4(a), according to the topological analysis, the top 8 compounds were identified as the key active compounds: *α*-eudesmol (degree = 73), *γ*-eudesmol (degree = 72), (−)-terpinen-4-ol (degree = 71), terpineol (degree = 71), hinesol (degree = 70), linalool (degree = 58), borneol (degree = 56), and *β*-eudesmol (degree = 51). Moreover, all the key active compounds correspond to more than 30 targets, suggesting that these compounds may have multitarget synergistic effects.

### 3.4. Protein-Protein Interaction (PPI) Network of Compound-AP Targets

As shown in Figure 4(b), there were 142 nodes and 710 interaction lines in the PPI network diagram with an average node degree of 10 and average aggregation coefficient of 0.458. The darker the color and the wider the area of the node in the diagram indicate the more significant the node. According to the degree value, the top 8 targets (TNF, IL-1*β*, PPAR*γ*, PPAR*α*, PTGS2, NCOA1, CNR1, and ESR1) of the PPI network were identified as the core therapeutic targets with the degree value greater than or equal to 24, suggesting that these targets may play a crucial role in the network of MVO in AP treatment. Thus, the interaction behaviors between these 8 core therapeutic targets and their corresponding compounds were further evaluated by using molecular docking simulation.

### 3.5. GO and KEGG Pathway Enrichment Analysis

**Figure 5:**
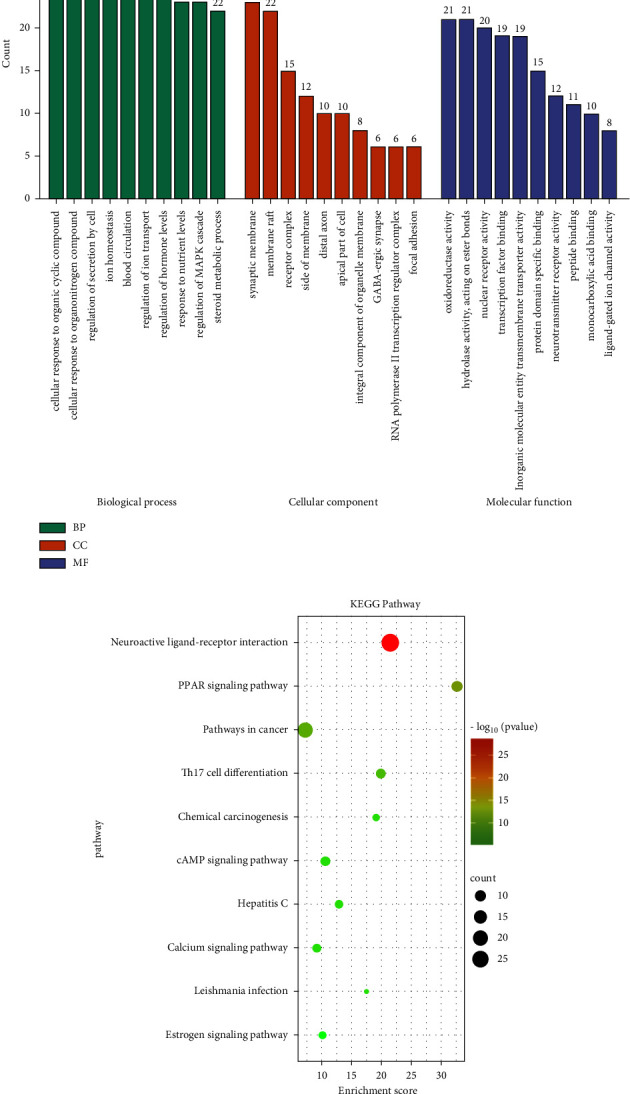
(a) GO enrichment analysis. The top 10 terms of BP, CC, and MF are shown. (b) KEGG pathway analysis. The sizes of the bubbles are proportional to the number of potential targets. The colors of the bubbles are illustrated from red to green in descending order of *p* values.

142 overlapping targets were introduced into Metascape for enrichment analysis. According to the GO enrichment results, the first 10 biological processes (BPs), cellular component (CC), and molecular function (MF) were selected for visualization, as shown in Figure 5(a) (*p* value <0.05). For biological processes, the overlapping targets were mainly enriched in functions associated with response to organic cyclic compound, steroid metabolic process, ion homeostasis, regulation of MAPK signaling pathway, and so on. From the KEGG pathway results, the pathway with the most enriched genes is the neuroactive ligand-receptor interaction pathway (with 28 targets), then followed by pathways in cancer (with 20 targets), PPAR signaling pathway (with 11 targets), Th17 cell differentiation pathway (with 10 targets), cAMP signaling pathway (with 10 targets), calcium signaling pathway (with 9 targets), chemical carcinogenesis pathway (with 8 targets), hepatitis C (with 8 targets), estrogen signaling pathway (with 7 targets), and leishmania infection (with 6 targets) (Figure 5(b)).

### 3.6. Compound-Target-Pathway Network

**Figure 6:**
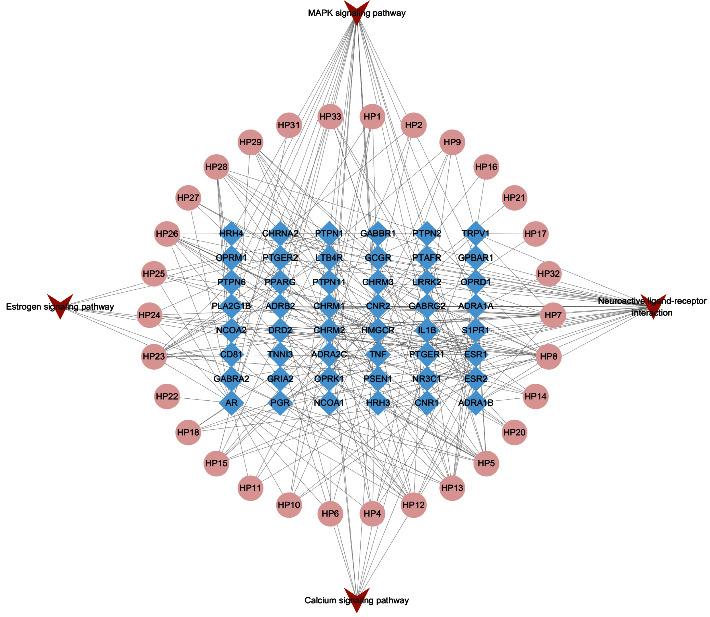
Compound-target-pathway network.

To simplify the analysis, pathways directly relating to AP treatment mechanism, including neuroactive ligand-receptor interaction, estrogen, MAPK, and calcium signaling pathway were constructed into a compound-target-pathway network. Those 4 pathways were associated with 48 targets (including 6 core therapeutic targets) and 30 potential active components (including 8 key active components) as shown in Figure 6.

### 3.7. Molecular Docking Simulation Analysis

**Figure 7:**
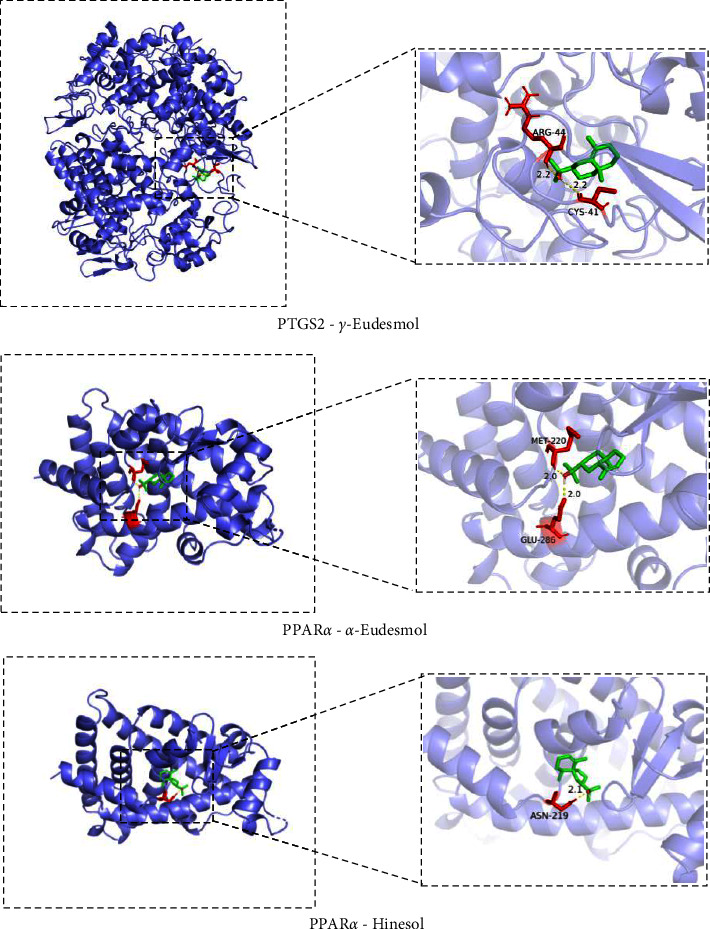
Molecular docking models of core therapeutic targets and key active compounds.

In this study, molecular docking simulation was applied to evaluate the binding activity between 8 core therapeutic targets (TNF, IL-1*β*, PPAR*γ*, PPAR*α*, PTGS2, NCOA1, CNR1, and ESR1) and their corresponding compounds of MVO. More negative of the docking energy indicates the greater binding ability among the compounds and the active site of the targets. Docking results are shown in Table 2, a total of 51 pairs of core therapeutic targets (*n* = 8); their corresponding compounds of MVO (*n* = 25) complexes were investigated using molecular docking simulation. Most of the binding complexes displayed a relatively strong binding affinity, and the average binding energies of them are −6.16 kcal/mol, which suggested that the potential active compounds of MVO had better binding activity with the core therapeutic targets. Among them, 19 pairs belonged to the core therapeutic targets-key active compounds complexes, and the binding energies of which were from −4.56 kcal/mol to −7.42 kcal/mol. The top 3 core therapeutic targets-key active compounds docking models are shown in Figure 7, including PTGS2-*γ*-eudesmol (−7.42 kcal/mol), PPAR*α*-*α*-eudesmol (−7.32 kcaJ/mol), and PPAR*α*-hinesol (−7.02 kcaJ/mol).

## 4. Discussion

Recently, more and more research studies have focused on TCM-based drug discovery and development to treat disease with complicated pathogenesis such as AP. In addition, the network pharmacology and molecular docking simulation have played a considerable role in the prediction of therapeutic mechanism in this process. In this paper, first, the compounds in MVO were analyzed by GC-MS. Then, a range of networks, including sample-compound-target network, PPI network of compound-AP targets, and compound-target-pathway network were constructed to reveal the mechanism of MVO in AP treatment by network pharmacology analyses. Finally, potential intermolecular interactions between the core therapeutic targets and their corresponding compounds based on network results were evaluated by molecular docking simulation.

The types and amount of compounds in MVO were various with different origins, harvesting time, or processing conditions [48–50]. Therefore, information of the compound in MVO obtained from few online databases or the GC-MS analysis results of a single sample of MOC would not be representative and universality and might lead to a bias in the result of network pharmacology analysis. To solve this problem, in this paper, 20 batches of MOC were collected from the main production regions, such as Sichuan, Hubei, Chongqing, and Zhejiang, and the compounds in the volatile oil of MOC were analyzed by GC-MS. 33 compounds that appeared in at least 18 of 20 samples were identified, representing 87.51%–96.41% of the total oil content. Among them, sesquiterpenes and oxygenated sesquiterpenes such as caryophyllene, caryophyllene oxide, calarene, *α*-, *β*-, and *γ*-eudesmol are abundantly in MVO, accounting for 0.58–13.43, 2.02–10.14, 1.93–4.63, 14.52–25.99, 10.83–23.07, and 6.50–14.56%, respectively, which was consistent with the previous studies [51, 52]. Therefore, those 33 compounds could relatively comprehensively and effectively reflect on the characteristic of the inner main compounds of MVO and be used for further network pharmacology analyses.

According to the sample-compound-target network, 8 active compounds including *α*-eudesmol (HP28), *γ*-eudesmol (HP23), (−)-terpinen-4-ol (HP8), terpineol (HP12), hinesol (HP26), linalool (HP5), borneol (HP13), and *β*-eudesmol (HP29) interacted with a quantity of AP-related targets, suggesting that these compounds played an important role in AP treatment. Previous study indicated that borneol exerted anti-AP effect in cerulein-induced AP mice model through reducing inflammation and oxidative stress [23]. However, the rest of them have not yet been confirmed to show any effect on the AP models or been applied in the AP treatment. In fact, lots of evidences showed that *β*-eudesmol and linalool have obvious anti-inflammatory, analgesic, antibacterial, and gastro-protective activities, suggesting that those two compounds may also have potential to treat AP by both alleviating the symptoms and preventing complications of this disease [53–56].

Based on the network topological analysis, GO and KEGG analysis, there were 8 targets (TNF, IL-1*β*, PPAR*γ*, PPAR*α*, PTGS2, NCOA1, CNR1, and ESR1) and 4 signaling pathways (neuroactive ligand-receptor interaction, estrogen, MAPK, and calcium signaling pathway) which have a very close association with the MVO in AP treatment.

The acute inflammatory response is one of the major pathological features of AP, leading to both the aggravation of local pancreatic damage and multiorgan failure of AP patient [57]. In the present study, there were three pathways (neuroactive ligand-receptor interaction, estrogen, and MAPK signaling pathway) which were associated with anti-inflammatory, involving a total of 30 compounds and 47 corresponding targets (including 6 core targets: CNR1, NCOA1, ESR1, PPAR*γ*, TNF, and IL-1*β*).

The neuroactive ligand-receptor interaction signaling pathway is a collection of all receptors and ligands related to intracellular signaling pathways and located on the plasma membrane [58]. Studies have shown that activation of multiple receptors in this signaling pathway such as *α*-, *β*-adrenergic receptor, dopamine receptor, or cholinergic receptors exerts anti-inflammatory effects in AP [59].

NCOA1, ESR1, and ESR2 are enriched in the estrogen signaling pathway. Studies have shown that NCOA1 is involved in the acute inflammatory process of AP through the regulation of IL-17 [60]. Both ESR1 and ESR2 can mediate anti-inflammatory actions. Estradiol may prevent anti-inflammatory process in the pancreas tissue via ESR1. The agonists of ESR1 and ESR2 may reduce oxidative stress, inflammatory, and pancreatic damage in the PBDL induced AP model [61].

As key signaling pathways of inflammatory diseases such as AP, MAPK signaling pathway regulates the transcription of inflammatory factors such as IL-1*β*, IL-6, TNF-*α,* and NF-*κ*B [62–64]. It was reported that MAPK signaling pathway has a high level of participation the inflammatory reaction in the development of AP from local to systemic and is considered as a potential target for AP treatment [64, 65].

TNF-*α* and IL-1*β* stimulate the production of other inflammatory factors such as IL-8 and IL-6 and are regarded as the most prominent “First line” cytokines in AP [66]. TNF-*α* promotes both neutrophils and macrophages to accumulate in the pancreas, which leads to aggravate the local pancreatic inflammation and develop as a systemic inflammatory response [67]. Moreover, TNF-*α* could additionally interact with IL-1*β* to further induce or aggravate organ injury [66]. It was reported that both the survival state and survival ratio of rats with necrotizing pancreatitis were improved after injection of TNF-*α* antibody [68].

PTGS2 also known as COX-2, plays a key role in the development and severity of AP [69, 70], which causes the release and activation of a large number of digestive enzymes in the pancreas as well as the disorders of pancreatic microcirculation, resulting in severe pathophysiological changes in the pancreas [71]. Mice that lack COX-2 genes have been reported to significantly decrease the severity of pancreatitis and pancreatitis-associated lung injury [69, 70]. Selective inhibitors of COX-2 were found to reduce pancreatic injury in AP [71].

Sustained elevated levels of Ca^2+^ in pancreatic cells are a marker for AP pathogenesis [72]. This overload Ca^2+^ can lead to disorders of acinar cells secretion, oxidative stress, impaired microcirculation, early activation of zymogens, mitochondrial dysfunction, and cell death, which together mediate the development of AP [73]. Currently, inhibition of intracellular Ca^2+^ overload has been considered as one of the most promising therapeutic approaches for AP treatment [74]. In this paper, a total of 9 therapeutic targets were mapped onto the calcium signaling pathway, and those 9 targets corresponded to 20 compounds of MVO, including 6 key compounds: (−)-terpinen-4-ol, terpineol, hinesol, *α*-, *β*-, and *γ*-eudesmol.

Through molecular docking simulation, it was found that the core therapeutic targets displayed good docking affinity for most of their corresponding compounds of MVO with an average binding energy of −6.16 kcal/mol. Notably, three eudesmol isoforms (*α*-, *β,* and *γ*-eudesmol) have high binding activity to the core therapeutic targets such as PPAR*γ*, PPAR*α*, PTGS2, or/and ESR1. Moreover, those three isoforms are also present at high levels in MVO, and the sum of relative content of them ranges from 31.85 to 63.62%. Consequently, we speculate that *α*-, *β*-, and *γ*-eudesmol may play a significant role in AP treatment of MVO.

In this paper, we found that the therapeutic mechanism of MVO in AP treatment has the characteristics of multicomponents and multitargets, which mainly involved in 8 key active compounds, 8 core therapeutic targets, and 4 signaling pathway, and associated with anti-inflammatory and inhibition of intracellular Ca^2+^ overload. The abovementioned study preliminarily revealed the active compounds and the potential mechanism of action of MVO in AP treatment, which provided enlightening information and basis for further exploration.

## 5. Conclusion

In this paper, the integrated strategy of GC-MS, network pharmacology, and molecular docking simulation was used to explore the active compounds as well as therapeutic mechanism of MVO in AP treatment. The results showed that 8 key active compounds may play pivotal roles in both anti-inflammatory and inhibition of intracellular Ca^2+^ overload effects in AP treatment by acting primarily on TNF, IL-1*β*, PPAR*γ*, PPAR*α*, PTGS2, NCOA1, CNR1, and ESR1 and involving in neuroactive ligand-receptor interaction, estrogen, MAPK, and calcium signaling pathway. Upon the abovementioned findings, MVO may be a potential “multicomponents” and “multitargets” agent for AP treatment. Our research will be useful for developing a novel TCM-based drug for clinical AP treatment. However, further extensive experiments are still necessary to verify the therapeutic mechanism of MVO in AP treatment.

## Figures and Tables

**Table 1:** 33 potential active compounds of MVO.

Nos.	Retention time	Compound	Molecular weight	Named as	Molecular formula	S1	S2	S3	S4	S5	S6	S7	S8	S9	S10	S11	S12	S13	S14	S15	S16	S17	S18	S19	S20
1	6.016	D-Limonene	136.23	HP1	C_10_H_16_	0.26	1.95	2.91	5.75	6.10	0.77	4.60	2.50	2.18	2.94	0.80	0.36	2.88	1.24	0.00	1.27	0.97	2.33	9.03	1.35
2	6.234	*β*-Phellandrene	136.23	HP2	C_10_H_16_	0.20	1.31	0.95	2.32	3.92	0.17	2.27	2.14	0.88	1.76	0.46	0.17	3.27	0.66	0.00	0.49	0.49	1.24	2.85	0.71
3	7.692	O-Cymene	134.22	HP3	C_10_H_14_	0.84	4.34	3.94	8.96	11.79	0.26	7.63	6.16	3.30	6.45	2.21	0.72	6.01	3.32	0.00	1.88	1.86	3.96	14.39	2.78
4	11.528	Copaene	204.35	HP4	C_15_H_24_	0.19	0.77	1.12	1.25	1.21	3.69	0.62	0.68	0.81	0.76	0.51	0.79	1.41	0.54	0.92	0.31	0.36	1.28	0.58	0.68
5	12.616	Linalool	154.25	HP5	C_10_H_18_O	0.94	1.50	4.95	0.62	3.83	0.78	0.68	1.80	4.35	1.84	0.72	0.99	1.92	1.04	0.07	0.52	1.17	2.62	4.48	1.52
6	13.186	(−)-Bornyl acetate	196.29	HP6	C_12_H_20_O_2_	1.50	1.20	0.23	0.23	0.49	0.16	0.67	1.89	0.54	2.27	2.23	1.01	1.50	1.35	0.21	0.00	0.86	1.59	0.87	1.00
7	13.469	Caryophyllene	204.35	HP7	C_15_H_24_	2.04	3.22	1.33	3.96	3.79	7.45	3.79	2.49	3.66	2.47	0.92	2.75	6.45	2.04	1.99	13.43	2.54	5.24	0.58	2.72
8	13.663	(−)-Terpinen-4-ol	154.25	HP8	C_10_H_18_O	0.17	0.26	0.36	0.46	1.01	0.16	0.34	0.40	0.36	0.27	0.17	0.19	0.31	0.18	0.00	0.00	0.16	0.31	0.40	0.22
9	15.027	*α*-Humulene	204.35	HP9	C_15_H_24_	0.98	1.49	1.01	0.96	1.58	1.69	1.43	0.88	2.14	1.23	0.54	1.53	2.86	0.80	1.90	5.13	1.09	2.05	0.27	1.35
10	15.168	2-Isopropenyl-4a,8-dimethyl-1,2,3,4,4a,5,6,7-octahydronaphthalene	204.35	HP10	C_15_H_24_	0.19	0.29	0.48	0.27	0.50	0.33	0.29	0.21	0.30	0.28	0.31	0.40	0.37	0.31	0.93	0.42	0.36	0.40	0.46	0.33
11	15.474	*γ*-Muurolene	204.35	HP11	C_15_H_24_	0.18	0.49	0.77	0.63	0.72	2.80	0.34	0.42	0.57	0.44	0.39	0.76	0.80	0.44	1.33	0.21	0.34	0.78	0.50	0.38
12	15.839	Terpineol	154.25	HP12	C_10_H_18_O	0.33	0.53	2.02	0.95	3.03	0.49	0.86	0.91	0.95	0.63	0.38	0.37	0.45	0.44	0.11	0.26	0.43	0.75	0.80	0.42
13	15.945	Borneol	154.25	HP13	C_10_H_18_O	0.87	0.75	1.06	0.22	0.69	0.17	0.66	1.46	0.71	0.89	1.19	0.47	1.60	1.21	0.00	0.00	0.66	0.97	0.37	0.79
14	16.221	(−)-*β*-Selinene	204.35	HP14	C_15_H_24_	1.14	1.79	2.33	1.41	2.04	2.09	1.39	1.15	1.84	1.68	1.83	2.23	2.21	1.52	4.69	2.59	1.84	2.11	1.96	1.92
15	16.339	*α*-Selinene	204.35	HP15	C_15_H_24_	1.00	2.33	2.61	1.58	2.61	0.00	1.48	1.33	2.22	1.91	1.60	2.68	3.07	1.56	5.59	2.91	1.82	2.50	2.34	2.17
16	17.268	(+)-delta-Cadinene	204.35	HP16	C_15_H_24_	0.69	1.44	2.00	2.63	1.85	4.95	1.47	1.11	1.78	1.13	0.64	2.09	2.84	1.15	3.06	0.41	0.79	2.50	0.80	1.49
17	17.539	3-Isopropyl-2-cyclopenten-1-one	124.18	HP17	C_8_H_12_O	0.94	0.51	0.71	0.56	0.15	0.28	0.37	0.55	0.40	0.59	1.64	0.82	0.38	1.29	0.73	0.17	1.09	0.41	0.40	0.65
18	19.456	Calamenene	202.33	HP18	C_15_H_22_	0.30	0.39	0.56	0.40	0.48	1.20	0.22	0.34	0.43	0.40	0.47	0.55	0.42	0.44	0.56	0.00	0.37	0.48	0.22	0.40
19	22.133	(1S)-4,7-Dimethyl-1-propan-2-yl-1,2-dihydronaphthalene	200.32	HP19	C_15_H_20_	0.29	0.41	0.66	0.57	0.56	1.95	0.34	0.42	0.56	0.40	0.37	0.57	0.35	0.44	0.72	0.00	0.38	0.50	0.45	0.44
20	24.268	Caryophyllene oxide	220.35	HP20	C_15_H_24_O	5.44	6.36	4.67	3.72	2.91	7.03	3.80	6.62	10.14	5.68	6.17	5.21	3.23	5.04	3.20	2.02	6.56	5.69	4.36	7.86
21	25.450	Santolina triene	136.23	HP21	C_10_H_16_	0.34	0.36	0.51	0.24	0.27	0.25	0.28	0.29	0.58	0.33	0.46	0.41	0.19	0.33	0.73	0.25	0.54	0.34	0.00	0.51
22	26.280	Humulene epoxide II	220.35	HP22	C_15_H_24_O	2.30	2.38	2.49	1.34	1.02	1.11	1.60	2.08	3.91	2.20	2.83	2.25	0.92	1.97	2.45	0.90	2.93	1.81	1.13	3.10
23	31.744	*γ*-Eudesmol	222.37	HP23	C_15_H_26_O	14.56	11.10	10.09	9.93	6.50	7.58	12.02	10.05	8.98	10.93	11.06	13.09	11.11	11.92	12.94	10.79	12.09	9.86	7.10	11.28
24	32.003	Calarene	204.35	HP24	C_15_H_24_	3.43	3.27	2.47	2.67	1.93	3.12	3.01	3.40	2.68	3.09	3.17	3.15	2.88	3.61	3.53	4.63	3.46	3.09	2.09	3.36
25	32.297	Aristolene	204.35	HP25	C_15_H_24_	2.04	1.77	1.58	1.57	1.29	1.52	1.69	1.74	1.55	1.66	2.16	1.83	1.44	2.08	2.08	2.20	2.14	1.63	1.13	1.92
26	32.456	Hinesol	222.37	HP26	C_15_H_26_O	1.50	1.39	1.07	1.19	0.91	1.34	1.33	1.43	1.16	1.31	1.46	1.36	1.18	1.54	1.48	1.87	1.49	1.30	0.87	1.44
27	33.014	*α*-Cubebene	204.35	HP27	C_15_H_24_	0.41	0.61	0.63	0.22	0.16	1.54	0.00	0.51	0.77	0.39	0.82	1.15	0.46	0.72	1.01	0.00	0.56	0.62	0.81	0.43
28	33.738	*α*-Eudesmol	222.37	HP28	C_15_H_26_O	25.99	21.77	18.60	19.86	14.52	16.66	22.32	21.05	18.49	21.62	20.84	22.40	17.94	22.27	22.53	23.00	22.71	19.15	15.15	22.07
29	34.067	*β*-Eudesmol	222.37	HP29	C_15_H_26_O	23.07	17.69	16.27	17.14	10.83	11.58	18.53	17.43	14.95	17.83	22.13	19.33	13.92	20.69	18.10	17.39	20.61	14.93	13.68	18.03
30	34.714	Hexamethylbenzene	162.27	HP30	C_12_H_18_	1.43	0.57	0.86	0.85	0.36	0.35	0.62	0.68	0.35	0.70	2.28	1.14	0.26	1.58	0.57	0.20	1.48	0.35	0.70	0.62
31	34.850	Neointermedeol	222.37	HP31	C_15_H_26_O	0.95	0.72	0.95	0.36	0.51	0.35	0.44	0.56	0.79	0.62	0.89	0.94	0.40	0.85	0.82	0.48	0.96	0.63	0.87	0.79
32	36.679	11,11-Dimethyl-4,8-dimethylenebicyclo[7.2.0]undecan-3-ol	220.35	HP32	C_15_H_24_O	0.66	0.39	0.46	0.33	0.50	0.46	0.35	0.35	0.43	0.29	0.86	0.58	0.00	0.56	0.34	0.31	0.70	0.25	0.28	0.45
33	38.308	—	220.35	—	C_15_H_24_O	1.06	0.59	0.91	0.68	1.16	0.79	0.70	0.48	0.67	0.44	1.44	0.93	0.25	0.91	0.70	0.80	1.25	0.44	0.41	0.74
34	39.896	—	220.35	—	C_15_H_24_O	1.07	0.60	0.64	0.38	0.52	0.50	0.44	0.56	0.77	0.46	1.04	0.68	0.16	0.69	0.36	0.35	0.85	0.41	0.50	0.74
35	48.590	((8R,8aS)-8-Isopropyl-5-methyl-3,4,6,7,8,8a-hexahydronaphthalen-2-yl)methanol	220.35	HP33	C_15_H_24_O	1.24	1.38	1.69	1.38	1.64	5.23	0.79	1.55	1.09	1.01	0.79	2.38	1.06	2.03	0.60	0.15	0.68	1.92	0.81	0.90

Total percentages of 23 common compounds						91.7	81.62	79.01	74.31	62.80	81.71	78.53	77.83	82.33	78.60	84.14	87.42	74.44	84.75	85.09	88.23	87.22	79.35	59.63	84.31
Relative contents of the 33 identified characteristic peaks						96.41	94.73	92.34	94.53	89.70	87.51	96.23	94.58	93.85	96.00	93.30	94.67	94.09	95.16	93.19	94.19	94.49	93.59	90.73	94.08

**Table 2:** 51 pairs molecular docking results.

Numbers	Core targets	PDB ID	Compounds	Docking affinity (kcal/mol)	Grid box	
					Size	Center
1	TNF-*α*	5M2J	*α*-Humulene	−4.89	*x* = 60, *y* = 60, *z* = 60, spacing = 1.000 Å	*x* = −7.851, *y* = −31.059, *z* = 1.722

2	IL-1*β*	1T4Q	*α*-Humulene	−5.30	*x* = 60, *y* = 60, *z* = 60, spacing = 0.775 Å	*x* = −15.992, *y* = 13.568, *z* = −1.681

3	PPAR*γ*	2VST	*γ*-Eudesmol	−6.35	*x* = 74, *y* = 74, *z* = 74, spacing = 1.000 Å	*x* = 22.268, *y* = 0.481, *z* = 25.314

4	PPAR*α*	3VI8	D-Limonene	−5.46	*x* = 68, *y* = 60, *z* = 78, spacing = 0.792 Å	*x* = 5.938, *y* = 2.655, *z* = 1.383
			O-Cymene	−5.08		*x* = 5.533, *y* = 1.987, *z* = 1.187
			Copaene	−6.70		*x* = 5.537, *y* = 1.486, *z* = 1.719
			Caryophyllene	−6.80		*x* = 5.845, *y* = 1.458, *z* = 1.634
			(−)-Terpinen-4-ol	−5.17		*x* = 5.845, *y* = 1.569, *z* = 1.831
			*α*-Humulene	−6.62		*x* = 5.928, *y* = 1.542, *z* = 1.884
			2-Isopropenyl-4a,8-dimethyl-1,2,3,4,4a,5,6,7-octahydronaphthalene	−6.71		*x* = 5.761, *y* = 1.486, *z* = 1.495
			*γ*-Muurolene	−6.61		*x* = 5.817, *y* = 1.514, *z* = 1.412
			Terpineol	−6.04		*x* = 5.761, *y* = 1.486, *z* = 1.466
			*α*-Selinene	−6.52		*x* = 5.817, *y* = 1.514, *z* = 1.439
			(+)-delta-Cadinene	−6.64		*x* = 5.761, *y* = 1.486, *z* = 1.467
			Santolina triene	−4.44		*x* = 5.705, *y* = 1.514, *z* = 1.522
			*γ*-Eudesmol	−6.83		*x* = 5.733, *y* = 1.514, *z* = 1.467
			Calarene	−6.65		*x* = 5.761, *y* = 1.598, *z* = 1.464
			Aristolene	−6.41		*x* = 5.817, *y* = 1.458, *z* = 1.468
			Hinesol	−7.02		*x* = 5.817, *y* = 1.402, *z* = 1.466
			*α*-Cubebene	−6.35		*x* = 5.789, *y* = 1.486, *z* = 1.411
			*α*-Eudesmol	−7.32		*x* = 5.733, *y* = 1.402, *z* = 1.523

5	PTGS2	5F19	D-Limonene	−5.64	*x* = 80, *y* = 80, *z* = 104, spacing = 1.000 Å	*x* = 22.208, *y* = 39.454, *z* = 39.308
			Linalool	−4.73		*x* = 22.851, *y* = 38.251, *z* = 39.588
			Caryophyllene	−6.99		*x* = 23.043, *y* = 37.898, *z* = 40.118
			(−)-Terpinen-4-ol	−5.79		*x* = 22.516, *y* = 38.476, *z* = 39.783
			*α*-Humulene	−6.05		*x* = 22.851, *y* = 37.092, *z* = 38.471
			Borneol	−5.20		*x* = 22.488, *y* = 38.539, *z* = 39.364
			(−)-*β*-Selinene	−6.97		*x* = 22.712, *y* = 37.832, *z* = 39.672
			Caryophyllene oxide	−6.40		*x* = 22.684, *y* = 37.953, *z* = 39.922
			*γ*-Eudesmol	−7.42		*x* = 22.286, *y* = 37.818, *z* = 39.644

6	NCOA1	7JYM	D-Limonene	−4.98	*x* = 72, *y* = 72, *z* = 60, spacing = 0.797 Å	*x* = −22.593, *y* = −2.282, *z* = −19.374
			(−)-Terpinen-4-ol	−4.56		*x* = −22.702, *y* = −2.565, *z* = −19.514

7	CNR1	5XR8	Calamenene	−6.82	*x* = 60, *y* = 86, *z* = 104, spacing = 1.000Å	*x* = −43.851, *y* = −137.887, *z* = 289.696
			Hinesol	−5.78		*x* = −43.795, *y* = −137.545, *z* = 289.696
			(8R,8aS)-8-Isopropyl-5-methyl-3,4,6,7,8,8a-hexahydronaphthalen-2-yl)methanol	−6.81		*x* = −43.795, *y* = −137.187, *z* = 289.752

8	ESR1	1A52	Copaene	−6.66	*x* = 80, *y* = 76, *z* = 80, spacing = 1.000 Å	*x* = 96.864, *y* = 14.815, *z* = 84.973
			(−)-Terpinen-4-ol	−4.97		*x* = 97.114, *y* = 14.243, *z* = 84.917
			2-Isopropenyl-4a,8-dimethyl-1,2,3,4,4a,5,6,7-octahydronaphthalene	−6.53		*x* = 97.002, *y* = 15.549, *z* = 85.084
			*γ*-Muurolene	−6.51		*x* = 97.002, *y* = 15.762, *z* = 84.054
			Terpineol	−4.90		*x* = 97.002, *y* = 13.277, *z* = 85.973
			Borneol	−5.04		*x* = 97.002, *y* = 14.811, *z* = 85.084
			*α*-Selinene	−6.65		*x* = 97.087, *y* = 14.324, *z* = 85.084
			Calamenene	−6.37		*x* = 97.309, *y* = 14.074, *z* = 85.084
			*γ*-Eudesmol	−6.49		*x* = 97.228, *y* = 15.758, *z* = 84.194
			Calarene	−6.68		*x* = 97.474, *y* = 15.181, *z* = 85.167
			Aristolene	−6.62		*x* = 97.421, *y* = 14.337, *z* = 85.084
			Hinesol	−6.77		*x* = 97.086, *y* = 14.225, *z* = 85.084
			*α*-Cubebene	−6.40		*x* = 97.141, *y* = 14.795, *z* = 85.168
			*α*-Eudesmol	−6.76		*x* = 97.058, *y* = 14.151, *z* = 85.084
			*β*-Eudesmol	−6.63		*x* = 97.002, *y* = 14.964, *z* = 84.083
			(8R,8aS)-8-Isopropyl-5-methyl-3,4,6,7,8,8a-hexahydronaphthalen-2-yl)methanol	−6.31		*x* = 97.198, *y* = 14.478, *z* = 85.084

## Data Availability

The main data used to support the findings of this study are included within the article.

## References

[1] Li Y., Zhang J., Zou J. (2020). Evaluation of four scoring systems in prognostication of acute pancreatitis for elderly patients. *BMC Gastroenterology*.

[2] Gao B., Zhang X., Xue D., Zhang W. (2020). Effects of Egr1 on pancreatic acinar intracellular trypsinogen activation and the associated ceRNA network. *Molecular Medicine Reports*.

[3] S M., Yadav D. (2019). Global epidemiology and holistic prevention of pancreatitis. *Nature Reviews Gastroenterology & Hepatology*.

[4] Radulova-Mauersberger O., Belyaev O., Birgin E. (2020). Indikationen zur chirurgischen und interventionellen Behandlung der akuten Pankreatitis. *Zentralbl Chir*.

[5] Oskarsson V., Hosseini S., Discacciati A. (2020). Rising incidence of acute pancreatitis in Sweden: National estimates and trends between 1990 and 2013. *United European Gastroenterol J*.

[6] Polishchuk I., Halperin D., Algedafy A. (2020). Epidemiology of acute pancreatitis in southern Israel: a retrospective study. *The Israel Medical Association Journal*.

[7] Sgaramella L. I., Gurrado A., Pasculli A. (2020). Open necrosectomy is feasible as a last resort in selected cases with infected pancreatic necrosis: a case series and systematic literature review. *World Journal of Emergency Surgery*.

[8] Lee P. J., Papachristou G. I. (2019). New insights into acute pancreatitis. *Nature Reviews Gastroenterology & Hepatology*.

[9] Koutroumpakis E., Slivka A., Furlan A. (2017). Management and outcomes of acute pancreatitis patients over the last decade: a US tertiary-center experience. *Pancreatology*.

[10] Working group of IPA\APA (2013). IAP/APA evidence-based guidelines for the management of acute pancreatitis. *Pancreatology*.

[11] Li J., Zhang S., Zhou R., Zhang J., Li Z.-F. (2017). Perspectives of traditional Chinese medicine in pancreas protection for acute pancreatitis. *WJG*.

[12] Zhan L., Pu J., Hu Y. (2021). Uncovering the pharmacology of xiaochaihu decoction in the treatment of acute pancreatitis based on the network pharmacology. *BioMed Research International*.

[13] Yang C., Wang T., Chen J. (2021). Traditional Chinese medicine formulas alleviate acute pancreatitis: pharmacological activities and mechanisms. *Pancreas*.

[14] Yang G. H., Zhao W. X. (2018). Early traditional Chinese medicine treatment of acute pancreatitis and its mechanism. *Journal Of Clinical Hepatology*.

[15] Chinese Pharmacopoeia Commission (2020). *Pharmacopoeia of the People’s Republic of China*.

[16] Jing W. G., Du J., Wang J. Y., Sun X. B., Lan Q. S. (2018). Review on chemical constituents of Magnoliae officinalis cortex. *Modern Chinese Medicine*.

[17] Sun W. J., Chen Y. F., Li H. C., Gao L., Feng D. X. (2019). Effect of categorized formulas da chengqitang in clinical treatment of acute pancreatitis. *Chinese Journal Of Experimental Traditional Medical Formulae*.

[18] Cao D., Xu Z. H., Wang F. F. (2015). Chemical composition and anti - inflammatory effects of essential oil from houpo (Magnolia officinalis bark). *Chinese Journal Of Traditional Medical Science*.

[19] Hu X. G., Dai W. Q., Han B. (2012). Experimental research of fatigue resistance and antibacterial effects of volatile oils of three kinds of Chinese medicine. *Modern Journal Of Integrated Traditional Chinese And Western Medicine*.

[20] Tang F., Liu M. C., Zhang S. Y. (2019). Comparative study on chemical composition and antibacterial activity of essential oils from patchouli and Magnolia officinalis alone and combination. *Traditional Chinese Drug Research & Clinical Pharmacology*.

[21] Hui N., Wei Y. H., Chen M. (2011). Chemical composition and gastric emptying-promoting effect of Pingwei powder San and the volatile oil of its constituent herbs in rats. *Chinese Traditional Patent Medicine*.

[22] Biradar S., Bantal V. (2012). Screening of natural antioxidants by using L-arginine induced acute pancreatitis model. *International Journal of Drug Development & Research*.

[23] Bansod S., Chilvery S., Saifi M. A. (2021). Borneol protects against cerulein‐induced oxidative stress and inflammation in acute pancreatitis mice model. *Environmental Toxicology*.

[24] Hopkins A. L. (2007). Network pharmacology. *Nature Biotechnology*.

[25] Hopkins A. L. (2008). Network pharmacology: the next paradigm in drug discovery. *Nature Chemical Biology*.

[26] Zhang Y., Li X., Guo C., Dong J., Liao L. (2020). Mechanisms of Spica Prunellae against thyroid-associated Ophthalmopathy based on network pharmacology and molecular docking. *BMC Complement Med Ther*.

[27] Zhang M., Li P., Zhang S. (2021). Study on the mechanism of the danggui-chuanxiong herb pair on treating thrombus through network pharmacology and zebrafish models. *ACS Omega*.

[28] An L., Lin Y., Li L. (2020). Integrating network pharmacology and experimental validation to investigate the effects and mechanism of Astragalus flavonoids against hepatic fibrosis. *Frontiers in Pharmacology*.

[29] Zhou S., Jiang N., Zhang M. (2020). Analyzing active constituents and optimal steaming conditions related to the hematopoietic effect of steamed panax notoginseng by network pharmacology coupled with response surface methodology. *BioMed Research International*.

[30] Liu B., Zheng X., Li J. (2021). Revealing mechanism of Caulis Sargentodoxae for the treatment of ulcerative colitis based on network pharmacology approach. *Bioscience Reports*.

[31] Saeed M., Tasleem M., Shoib A. (2022). Identification of putative plant-based ALR-2 inhibitors to treat diabetic peripheral neuropathy. *Current Issues in Molecular Biology*.

[32] Zrieq R., Snoussi M., Algahtan F. D. (2022). Repurposing of anisomycin and oleandomycin as a potential anti-(SARS-CoV-2) virus targeting key enzymes using virtual computational approaches. *Cellular and Molecular Biology (Paris, France, Print)*.

[33] Tasleem M., Alrehaily A., Almeleebia T. M. (2021). Investigation of antidepressant properties of yohimbine by employing structure-based computational assessments. *Current Issues in Molecular Biology*.

[34] Alshahrani M. Y., Alshahrani K. M., Tasleem M. (2021). Computational screening of natural compounds for identification of potential anti-cancer agents targeting MCM7 protein. *Molecules*.

[35] TasleIsem M., hrat R., Islam A., Ahmad F., Hassan Md. (2014). Structural characterization, homology modeling and docking studies of ARG674 mutation in MyH8 gene associated with trismus-pseudocamptodactyly syndrome. *LDDD*.

[36] Xu J., Wang F., Guo J. (2020). Pharmacological mechanisms underlying the neuroprotective effects of alpinia oxyphylla miq. On alzheimer’s disease. *International Journal of Molecular Sciences*.

[37] Li Z., Li J., Zhang F. (2020). Antidiarrheal effect of sechang-zhixie-san on acute diarrhea mice and network pharmacology deciphering its characteristics and potential mechanisms. *Evid Based Complement Alternat Med*.

[38] Wu Y., Liu X., Li G. (2022). Integrated bioinformatics and network pharmacology to identify the therapeutic target and molecular mechanisms of Huangqin decoction on ulcerative Colitis. *Scientific Reports*.

[39] Dong K. F., Huo M. Q., Sun H. Y., Li T. K., Li D. Mechanism of Astragalus membranaceus in the treatment of laryngeal cancer based on gene co-expression network and molecular docking. *Scientific Reports*.

[40] Wang G., Zhou B., Wang Z. (2021). Pharmacological mechanisms underlying the anti-asthmatic effects of modified guomin decoction determined by network pharmacology and molecular docking. *Frontiers in Molecular Biosciences*.

[41] Cao X., You G., Li H. (2019). Comparative investigation for rotten xylem (kuqin) and strip types (tiaoqin) of scutellaria baicalensis georgi based on fingerprinting and chemical pattern recognition. *Molecules*.

[42] Costanzo G., Iesce M. R., Naviglio D. (2020). Comparative studies on different citrus cultivars: a revaluation of waste Mandarin components. *Antioxidants*.

[43] Zhang X., Zhao Y., Guo L. (2017). Differences in chemical constituents of Artemisia annua L from different geographical regions in China. *PLoS One*.

[44] Song S.-Y., Park D.-H., Seo S.-W. (2019). Effects of harvest time on phytochemical constituents and biological activities of panax ginseng berry extracts. *Molecules*.

[45] Cautela D., Vella F. M., Laratta B. (Oct. 2019). The effect of processing methods on phytochemical composition in bergamot juice. *Foods*.

[46] Oliveros J. C. (2007-2015). *Venny. An Interactive Tool for Comparing Lists with Venn’s Diagrams*.

[47] von Mering C., Jensen L. J., Snel B. (2005). STRING: known and predicted protein-protein associations, integrated and transferred across organisms. *Nucleic Acids Research*.

[48] Zhang C. X., Yang L. X., Yu X. (2009). Effects of tree ages and geographic area on quality of bark of Magnolia officinalis and Mofficinalis var biloba. *China Journal of Chinese Materia Medica*.

[49] Lin Y., Wang L., Zhang J., Yuan K. (2010). GC-MS analysis of chemical composition of Cortex Magnolia Officinalis Oil before and after processing. *Chinese Traditional and Herbal Drugs*.

[50] Fang X. P., Lu Y. S., Wu Q., Hu G. P. (2012). Comparative research on volatile oil components of Magnolia officinalis in guizhou different regions. *Chinese Journal Of Experimental Traditional Medical Formulae*.

[51] Yuan J. F., Qiao Y. H., Peng S. T. (2019). Analysis on volatile constituents in Magnolia officinals and different processed products by GC-MS. *Chinese Traditional Patent Medicine*.

[52] Bai J., Li X. R., Fan Z. B., Zhu L. F. (2011). Comparative analysis of volatile constituents in herbal pair citrus auruntium fructus–magnoliae officinalis cortex and its single herbs by GC-MS and AMWFA. *Research And Practice On Chinese Medicines*.

[53] Li Y., Lv O., Zhou F. (2015). Linalool inhibits LPS-induced inflammation in BV2 microglia cells by activating Nrf2. *Neurochemical Research*.

[54] Mohamed M. E., Abduldaium M. S., Younis N. S. (2021). Cardioprotective effect of linalool against isoproterenol-induced myocardial infarction. *The Life*.

[55] Li X.-J., Yang Y.-J., Li Y.-S., Zhang W. K., Tang H.-B. (2016). *α*-Pinene, linalool, and 1-octanol contribute to the topical anti-inflammatory and analgesic activities of frankincense by inhibiting COX-2. *Journal of Ethnopharmacology*.

[56] Acharya B., Chaijaroenkul W., Na-Bangchang K. (2021). Therapeutic potential and pharmacological activities of *β*-eudesmol. *Chemical Biology &amp;amp; Drug Design*.

[57] Zheng Z., Ding Y.-X., Qu Y.-X., Cao F., Li F. (2021). A narrative review of the mechanism of acute pancreatitis and recent advances in its clinical management. *Am J Transl Res*.

[58] Su S.-Y., Hsieh C.-L., Wu S.-L. (2009). Transcriptomic analysis of EGb 761-regulated neuroactive receptor pathway in vivo. *Journal of Ethnopharmacology*.

[59] Li F. J., Li Q. (2012). The role of neuroimmune regulation in acute pancreatitis. *Chinese Journal of Neuroimmunology and Neurology*.

[60] Zhu K., Zhang M., Long J., Zhang S., Luo H. (2021). Elucidating the mechanism of action of salvia miltiorrhiza for the treatment of acute pancreatitis based on network pharmacology and molecular docking Technology. *Computational and Mathematical Methods in Medicine*.

[61] Guleken Z., Ozbeyli D., Acikel-Elmas M. (2017). The effect of estrogen receptor agonists on pancreaticobiliary duct ligation induced experimental acute pancreatitis. *Journal of Physiology & Pharmacology*.

[62] Abozaid O. A. R., Moawed F. S. M., Ahmed E. S. A., Ibrahim Z. A. (2020). Cinnamic acid nanoparticles modulate redox signal and inflammatory response in gamma irradiated rats suffering from acute pancreatitis. *Biochimica et Biophysica Acta, Molecular Basis of Disease*.

[63] Han B., Zhou H., Jia G. (2016). MAPKs and Hsc70 are critical to the protective effect of molecular hydrogen during the early phase of acute pancreatitis. *FEBS Journal*.

[64] Irrera N., Bitto A., Interdonato M., Squadrito F., Altavilla D. (2014). Evidence for a role of mitogen-activated protein kinases in the treatment of experimental acute pancreatitis. *World Journal of Gastroenterology*.

[65] Escobar J., Pereda J., López-Rodas G., Sastre J. (Mar. 2012). Redox signaling and histone acetylation in acute pancreatitis. *Free Radical Biology and Medicine*.

[66] Velayutham R., Patil V. S., T S S. (2015). Study of biomedical efficacy of beta asarone against pancreatitis rats. *International Research Journal of Pharmacy*.

[67] El-Ashmawy N. E., Khedr N. F., El-Bahrawy H. A., Hamada O. B. (2018). Anti-inflammatory and antioxidant effects of captopril compared to methylprednisolone in L-arginine-induced acute pancreatitis. *Digestive Diseases and Sciences*.

[68] Oruc N., Ozutemiz A. O., Yukselen V. (Jan. 2004). Infliximab: a new therapeutic agent in acute pancreatitis?. *Pancreas*.

[69] Ethridge R. T., Chung D. H., Slogoff M. (2002). Cyclooxygenase-2 gene disruption attenuates the severity of acute pancreatitis and pancreatitis-associated lung injury. *Gastroenterology*.

[70] Song A. M., Bhagat L., Singh V. P. (2002). Inhibition of cyclooxygenase-2 ameliorates the severity of pancreatitis and associated lung injury. *American Journal of Physiology - Gastrointestinal and Liver Physiology*.

[71] Bu L. M., Tang C., Xu M. (2010). Advances in understanding the role ofcyclooxygenase-2 in the pathogenesis of acute pancreatitis. *World Chinese Journal of Digestology*.

[72] Maléth J., Hegyi P. (2016). Ca^2+^ toxicity and mitochondrial damage in acute pancreatitis: translational overview. *Philosophical Transactions of the Royal Society of London B Biological Sciences*.

[73] Pallagi P., Madácsy T., Varga Á., Maléth J. (2020). Intracellular Ca^2+^ signalling in the pathogenesis of acute pancreatitis: recent advances and translational perspectives. *International Journal of Molecular Sciences*.

[74] Waldron R. T., Lugea A., Pandol S. J. (2019). Brake adjustment: Ca^2+^ entry pathway provides a novel target for acute pancreatitis therapy. *Annals of Translational Medicine*.

